# Time-dependent anti-Müllerian hormone dynamics during GnRHa-based endocrine therapy in young breast cancer survivors: a longitudinal real-world cohort study

**DOI:** 10.1186/s13058-026-02359-9

**Published:** 2026-08-04

**Authors:** Chang Jiang, Xiwen Bi, Xin Hua, Zhongyu Yuan, Ying Guo, Shusen Wang, Yanxia Shi, Fei Xu, Yongyi Zhong, Cong Xue

**Affiliations:** 1 Department of VIP, State Key Laboratory of Oncology in South China, Guangdong Provincial Clinical Research Center for Cancer, Sun Yat-sen University Cancer Center, Guangzhou, 510060 People’s Republic of China; 2 Department of Medical Oncology, State Key Laboratory of Oncology in South China, Guangdong Provincial Clinical Research Center for Cancer, Sun Yat-sen University Cancer Center, Guangzhou, 510060 People’s Republic of China; 3 Department of Radiation Oncology, Guangdong Provincial People’s Hospital, Guangdong Academy of Medical Sciences, Southern Medical University, Guangzhou, 510100 People’s Republic of China; 4 Department of Clinical Research, State Key Laboratory of Oncology in South China, Guangdong Provincial Clinical Research Center for Cancer, Sun Yat-sen University Cancer Center, Guangzhou, 510060 People’s Republic of China

**Keywords:** Anti-Müllerian hormone, Young breast cancer, GnRHa-based endocrine therapy, Ovarian function, Fertility counseling, Survivorship

## Abstract

**Background:**

Anti-Müllerian hormone (AMH) is widely used to assess ovarian reserve in young women with breast cancer. However, its interpretation during gonadotropin-releasing hormone agonist (GnRHa)-based endocrine therapy remains challenging because pharmacologic ovarian suppression may alter AMH levels over time. We aimed to characterize longitudinal AMH dynamics according to chemotherapy exposure and subsequent GnRHa-based endocrine therapy.

**Methods:**

We conducted a retrospective longitudinal cohort study of women diagnosed with early breast cancer before age 40 at Sun Yat-sen University Cancer Center between August 2019 and December 2024. Patients were categorized into four groups according to chemotherapy exposure and GnRHa-based endocrine therapy. Longitudinal trajectories of log-transformed AMH were modeled using linear mixed-effects models with restricted cubic splines, adjusting for prespecified clinical covariates. Sensitivity analyses included baseline-AMH adjustment and exclusion of observations with extreme standardized residuals.

**Results:**

The full cohort included 685 women contributing 1758 AMH measurements. The adjusted complete-case model included 613 women contributing 1602 measurements. The group-by-time interaction was significant (likelihood ratio χ^2^ = 249.33, df = 9, *P* < 0.001), demonstrating distinct AMH trajectories across treatment groups. Among women without chemotherapy, predicted AMH did not differ significantly between those with and without GnRHa-based endocrine therapy. Among chemotherapy-treated patients, the GnRHa-based endocrine therapy group was associated with lower predicted AMH at 12 and 36 months, but the differences attenuated at later follow-up. Similar trajectory patterns were observed in sensitivity analyses.

**Conclusions:**

AMH trajectories during GnRHa-based endocrine therapy are time-dependent and influenced by treatment context. AMH measurements during GnRHa exposure should be interpreted in relation to treatment timing and longitudinal changes rather than as isolated indicators of ovarian reserve.

**Supplementary Information:**

The online version contains supplementary material available at https://doi.org/10.1186/s13058-026-02359-9.

## Introduction

Young women with breast cancer face distinct survivorship challenges that extend beyond cancer control. As treatment outcomes continue to improve, preservation of ovarian function and reproductive potential has become an increasingly important component of long-term care [1, 2]. Fertility-related concerns may influence treatment decisions, quality of life, and adherence to prolonged endocrine therapy, particularly among women with hormone receptor-positive disease receiving ovarian function suppression [3, 4].

Anti-Müllerian hormone (AMH) is widely used as a biomarker of ovarian reserve and is commonly incorporated into fertility counseling for young women before and after cancer treatment [5–7]. Unlike menstrual status or gonadotropins, AMH is less dependent on cycle phase and may provide early information regarding treatment-related ovarian injury [5, 6]. However, AMH after breast cancer treatment is not a static measurement [8–11]. Its level may vary according to age, baseline ovarian reserve, chemotherapy exposure, endocrine therapy, and the timing of blood sampling relative to treatment [6, 8, 12–16]. Therefore, single AMH values during survivorship may be difficult to interpret without longitudinal context.

This uncertainty is particularly relevant for young breast cancer survivors receiving gonadotropin-releasing hormone agonist (GnRHa)-based endocrine therapy. GnRHa suppresses the hypothalamic-pituitary-ovarian axis and is widely used either during chemotherapy for ovarian preservation or as adjuvant endocrine therapy combined with selective estrogen receptor modulators (SERMs) or aromatase inhibitors (AIs) [17–20]. Therefore, low AMH levels during GnRHa exposure may reflect pharmacologic ovarian suppression, chemotherapy-related ovarian injury, age-related decline, or a combination of these factors [7, 21, 22]. Distinguishing these possibilities is clinically important for fertility counseling and interpretation of ovarian reserve.

Previous studies have shown that chemotherapy induces rapid AMH decline, followed by variable recovery depending on age, treatment exposure, and baseline ovarian reserve [6, 8–13]. Randomized trials evaluating GnRHa during chemotherapy have primarily focused on ovarian function recovery, premature ovarian insufficiency, and fertility outcomes [17–20, 23–26]. In contrast, the longitudinal AMH pattern during subsequent GnRHa-based endocrine therapy remains poorly characterized. In particular, limited data are available regarding nonlinear AMH trajectories over extended follow-up, including early suppression, recovery, plateau, and later decline [13, 22, 27–29].

In this retrospective longitudinal cohort study, we characterized AMH trajectories in young women diagnosed with early breast cancer before age 40 according to chemotherapy exposure and subsequent GnRHa-based endocrine therapy. Using linear mixed-effects models with restricted cubic splines, we aimed to describe time-dependent AMH dynamics and to improve interpretation of AMH measurements during breast cancer survivorship.

## Patients and methods

### Study design and population

This retrospective longitudinal cohort study included women diagnosed with early breast cancer before age 40 at Sun Yat-sen University Cancer Center (SYSUCC) between August 2019 and December 2024. Eligible patients had histologically confirmed ductal carcinoma in situ (DCIS), lobular carcinoma in situ (LCIS), or invasive breast cancer, and at least one serum AMH measurement during follow-up. Patients with bilateral oophorectomy before or during follow-up were excluded. Endocrine therapy was not an exclusion criterion because the study specifically evaluated AMH dynamics during adjuvant endocrine treatment, including GnRHa-based ovarian function suppression.

The study was approved by the institutional review board of SYSUCC, approval number B2023-351-01, and was conducted in accordance with the Declaration of Helsinki and Good Clinical Practice guidelines. The requirement for individual informed consent was waived because of the retrospective nature of the study. Clinical trial number: not applicable.

### Data collection and treatment variables

Clinical and laboratory data were extracted from the electronic medical record system. Variables included age at diagnosis, body mass index (BMI), tumor and treatment characteristics, chemotherapy exposure, concurrent GnRHa use during chemotherapy, adjuvant endocrine therapy, anti-HER2 therapy, and all available AMH measurements during follow-up.BMI was defined as the value recorded at the first visit to SYSUCC before initiation of study follow-up and was categorized as < 25 or ≥ 25 kg/m^2^.

The date of breast cancer diagnosis was defined as the date of pathological confirmation. Follow-up time for each AMH measurement was calculated as the interval in months from diagnosis to the date of AMH testing. Follow-up was defined as the period from diagnosis to the date of each AMH measurement, disease recurrence, death, or the end of data collection.

### AMH measurement

AMH measurements were obtained from clinically performed laboratory tests during routine care rather than from centrally assayed stored serum samples. At SYSUCC, AMH assessment is routinely recommended at the initial oncology visit for young women before systemic treatment whenever feasible. Follow-up AMH testing is not protocol-mandated and is performed at the treating physician’s discretion, commonly for ovarian reserve assessment, fertility counseling, or endocrine follow-up. Fertility preservation and reproductive counseling are provided through a multidisciplinary referral pathway with our affiliated reproductive medicine center when clinically indicated.

Serum AMH levels were measured using an electrochemiluminescence immunoassay on a Cobas E801 analyzer (Roche Diagnostics, Mannheim, Germany) as previously described [30]. The lower limit of quantification was 0.01 ng/mL, and values below this threshold were assigned 0.01 ng/mL for analysis. Because AMH values showed a right-skewed distribution, a natural logarithmic transformation was applied before modeling [14, 16].

Baseline AMH was defined as the last AMH measurement obtained after pathological diagnosis but before initiation of systemic therapy. For patients receiving neoadjuvant chemotherapy, baseline AMH was defined before neoadjuvant chemotherapy. Patients without pretreatment AMH measurements were included in the primary longitudinal analysis using all available AMH measurements, with follow-up time indexed from the date of diagnosis. Baseline-adjusted analyses were performed as sensitivity analyses among patients with available baseline AMH.

### Exposure groups

To characterize AMH dynamics according to chemotherapy exposure and subsequent GnRHa-based endocrine therapy, patients were categorized into four mutually exclusive groups: No CT/No GnRHa-ET, No CT/GnRHa-ET, CT/No GnRHa-ET, and CT/GnRHa-ET.

Chemotherapy exposure was defined as receipt of at least one cycle of cytotoxic chemotherapy. Chemotherapy regimens were categorized as cyclophosphamide-based or non-cyclophosphamide-based. Anti-HER2 therapy was recorded as received or not received.

GnRHa exposure during chemotherapy was defined as initiation of at least one dose of GnRHa before or concurrently with chemotherapy and was considered separately from subsequent GnRHa-based endocrine therapy. GnRHa-based endocrine therapy was defined as adjuvant endocrine therapy containing GnRHa combined with either a SERM or an AI. Patients who switched from SERM to AI were classified in the AI group.

This grouping strategy was prespecified to distinguish chemotherapy exposure from longitudinal AMH patterns observed during GnRHa-based endocrine therapy.

### Statistics

Continuous variables were summarized as medians and interquartile ranges (IQRs) or ranges, as appropriate. Categorical variables were summarized as frequencies and percentages. Characteristics were compared across the four exposure groups using Kruskal–Wallis tests for continuous variables and Fisher’s exact tests or chi-square tests for categorical variables, as appropriate.

Longitudinal AMH trajectories were analyzed using linear mixed-effects models [27]. The outcome variable was natural log-transformed AMH. Time since diagnosis was modeled flexibly using restricted cubic splines with four knots to capture potential nonlinear patterns, including early decline, recovery, plateau, and later decline [13–15]. The primary exposure was the four-level treatment group. Group-specific trajectories were estimated by including interaction terms between treatment group and spline-transformed time. A random intercept for each patient was included to account for within-patient correlation from repeated AMH measurements. The contribution of group-by-time interaction terms was assessed using likelihood ratio tests comparing models with and without the interaction.

The primary multivariable model adjusted for clinically relevant covariates selected a priori, including age at diagnosis, BMI category (< 25 vs. ≥ 25 kg/m^2^), anti-HER2 therapy, and cyclophosphamide-based chemotherapy. Concurrent GnRHa use during chemotherapy was included as a binary covariate, with non-chemotherapy patients coded as unexposed. Endocrine therapy subtype was not included in the primary multivariable model to avoid collinearity because AI therapy was administered only in combination with GnRHa in this cohort.

Multivariable models were performed using complete cases for the covariates included in each model. The number of patients and AMH observations included in each sensitivity analysis is reported in the corresponding table or figure legend.

Predicted AMH trajectories were estimated from the fitted models and back-transformed for graphical presentation when appropriate. Pairwise comparisons between clinically relevant groups were performed at prespecified time points, 12, 36, 60 and 84 months, representing early treatment, intermediate follow-up, and long-term survivorship phases, with Tukey adjustment for multiple comparisons. Trajectories were truncated at 84 months because observations beyond this point were sparse and produced unstable estimates. The trajectory plots represent model-predicted AMH values derived from linear mixed-effects models fitted to all complete-case longitudinal observations, rather than observed mean values at each time point.

Sensitivity analyses included adjustment for baseline AMH among patients with available baseline AMH measurements, exclusion of observations with extreme standardized residuals, adjustments for chemotherapy-related treatment heterogeneity, and analyses restricted to patients with repeated AMH measurements.

Model assumptions were evaluated by visual inspection of residual-versus-fitted plots, quantile–quantile (Q–Q) plots of residuals, residuals over follow-up time, and observed-versus-fitted lnAMH values. The distribution of follow-up observations across time windows was summarized by treatment group to assess the stability of long-term trajectory estimates.

All analyses were performed using R version 4.4.3 (R Foundation for Statistical Computing). Statistical significance was defined as a two-sided *P* value < 0.05, except where adjusted *P* values were reported for post hoc comparisons.

## Results

### Basic characteristics

**Fig. 1:**
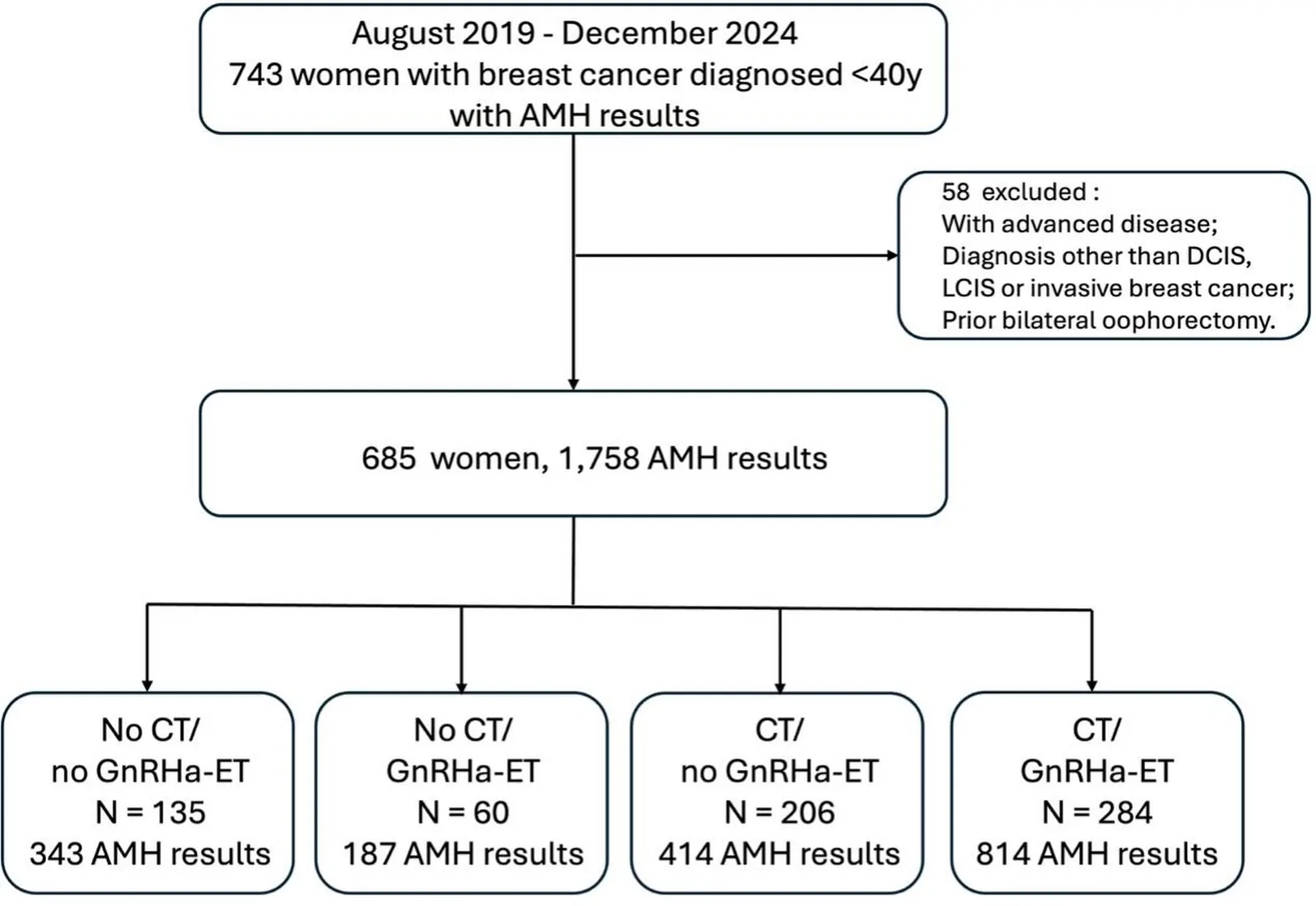
Flow chart

From August 2019 to December 2024, 685 women aged < 40 years at diagnosis with early breast cancer (EBC) underwent 1758 AMH measurements. The median number of AMH measurements per woman was 2 (range: 1–21); median time from diagnosis to AMH was 1.3 years (range: 0–13.1). A subset of 317 women (46.3%) had baseline AMH. The flow chart is presented in Fig. 1.

Baseline characteristics are summarized in Table 1. Median age at diagnosis was 35 years (range: 20–39);14.7% (N = 101) were aged < 30. In total, 490 women (71.5%) received chemotherapy. Per predefined criteria, the cohort was stratified into: No CT/No GnRHa-ET (N = 135), No CT/GnRHa-ET (N = 60), CT/No GnRHa-ET (N = 206), and CT/GnRHa-ET (N = 284). Treatment-related characteristics differed across groups, including concurrent GnRHa use during chemotherapy, anti-HER2 therapy, and cyclophosphamide-based chemotherapy (all *P* < 0.05).

**Table 1:** Patient characteristics

Characteristics	Total (N = 685)	No CT/No GnRHa-ET (N = 135)	No CT/GnRHa-ET (N = 60)	CT/No GnRHa-ET (N = 206)	CT/GnRHa-ET (N = 284)	*P*
Age at diagnosis (Median, range, years)	35 (20–39)	35 (20–39)	35 (23–39)	35 (23–39)	35 (20–39)	0.074
< 30	101 (14.7)	21 (15.6)	7 (11.7)	31 (15.0)	42 (14.8)	
30–34	210 (30.7)	36 (26.7)	18 (30.0)	62 (30.0)	94 (33.1)	
35–39	374 (54.6)	78 (57.7)	35 (58.3)	113 (55.0)	148 (52.1)	
BMI (kg/m^2^)						0.460
< 25	568 (85.9)	119 (90.8)	47 (83.9)	168 (84.8)	234 (84.8)	
≥ 25	93 (14.1)	12 (9.2)	9 (16.1)	30 (15.2)	42 (15.2)	
Missing	24	4	4	8	8	
Concurrent GnRHa during chemotherapy	122 (17.8)	0 (0)	0 (0)	35 (17.0)	87 (30.6)	< 0.001
Anti-HER2 therapy	131 (19.1)	0 (0)	0 (0)	66 (32.0)	65 (22.9)	< 0.001
Cyclophosphamide-based regimen	417 (60.9)	0 (0)	0 (0)	153 (74.3)	264 (93.0)	< 0.001
Endocrine therapy	526 (76.8)	113 (83.7)	60 (100)	69 (33.5)	284 (100)	< 0.001
SERM	245 (35.8)	113 (83.7)	20 (33.3)	69 (33.5)	43 (15.1)	
AI	281 (41.0)	0 (0)	40 (66.7)	0 (0)	241 (84.9)	
Baseline AMH (Median, IQR, ng/mL, N = 317)	2.4 (1.27, 3.94)	3.29 (1.68, 4.22)	2.22 (1.66, 4.10)	1.93 (0.99, 3.05)	2.48 (1.27, 3.90)	0.010

Percentages for BMI categories were calculated among patients with available BMI data

*AI* aromatase inhibitor, *BMI* body mass index, *CT* chemotherapy, *ET* endocrine therapy, *GnRHa* gonadotropin-releasing hormone agonist, *HER2* human epidermal growth factor receptor 2, *IQR* interquartile range, *SERM* selective estrogen receptor modulator

Among chemotherapy-treated patients, chemotherapy components and systemic treatment characteristics differed between groups (Table S1). Additional adjustment for these factors yielded trajectory patterns consistent with the primary analysis.

Overall, 240 of 1758 AMH measurements (13.7%) were below the lower limit of quantification and were assigned 0.01 ng/mL for analysis. At the patient level, 125 of 685 patients (18.2%) had at least one AMH measurement below the lower limit of quantification. The distribution of below-quantification AMH values by treatment group, chemotherapy exposure, and follow-up interval is shown in Table S2.

### Primary longitudinal AMH analysis

**Fig. 2:**
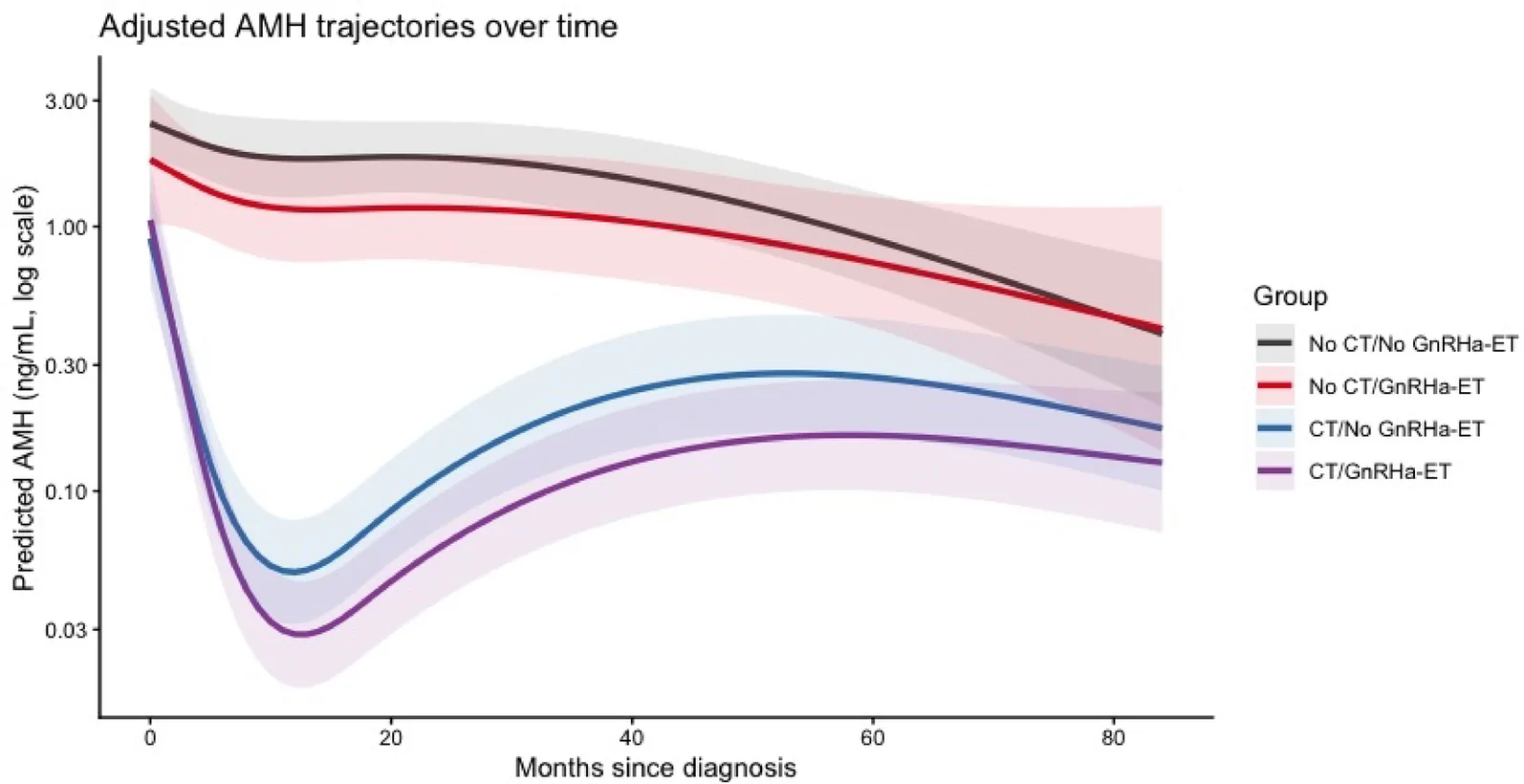
Adjusted predicted longitudinal AMH trajectories according to chemotherapy exposure and GnRHa-based endocrine therapy (613 patients; 1602 AMH observations). Predicted AMH values were derived from the primary multivariable linear mixed-effects model and back-transformed from lnAMH. The y-axis is displayed on a logarithmic scale. Shaded areas represent 95% confidence intervals. AMH, anti-Müllerian hormone; CT, chemotherapy; GnRHa-ET, gonadotropin-releasing hormone agonist-based endocrine therapy

The primary model was adjusted for age at diagnosis, BMI, concurrent GnRHa use during chemotherapy, cyclophosphamide-based chemotherapy, and anti-HER2 therapy. The group-by-time interaction significantly improved model fit compared with the model without interaction terms (likelihood ratio χ^2^ = 249.33, df = 9, *P* < 0.001); this indicates distinct longitudinal AMH trajectories across treatment groups. The adjusted trajectories are shown in Fig. 2.

In non-chemotherapy cohorts, the No CT/No GnRHa-ET group exhibited a gradual, age-concordant decline in predicted AMH over follow-up. Among chemotherapy-treated patients, both the CT/No GnRHa-ET and CT/GnRHa-ET groups showed a marked early decline in predicted AMH. The CT/GnRHa-ET group showed lower early predicted AMH levels, followed by attenuation of between-group differences over longer follow-up.

The numbers of unique patients and AMH measurements contributing to each follow-up interval are provided in Table S3. Observation density was highest within the first 12 months and declined over longer follow-up, particularly beyond 60 months.

### Post-hoc pairwise comparisons

In non-chemotherapy cohorts, predicted AMH levels did not differ significantly between the No CT/No GnRHa-ET and No CT/GnRHa-ET groups at any prespecified time point. The No CT/GnRHa-ET group exhibited lower early AMH—followed by gradual convergence toward the No CT/No GnRHa-ET trajectory—with no statistically significant divergence at any time (Table S4).

Among chemotherapy-treated patients, the CT/GnRHa-ET group had significantly lower predicted AMH than the CT/No GnRHa-ET group at 12 months (estimate for CT/No GnRHa-ET minus CT/GnRHa-ET: 0.566, adjusted *P* = 0.026) and 36 months (estimate 0.644, adjusted *P* = 0.023). Differences were no longer statistically significant at 60 months (estimate 0.435, adjusted *P* = 0.267) or 84 months (estimate 0.113, adjusted *P* = 0.988). Differences were attenuated at later follow-up time points (Table S5).

### Model diagnostics

Model diagnostic plots did not suggest major systematic misspecification. Residuals exhibited no marked time-dependent pattern, supporting the appropriateness of spline-based time modeling. Q–Q plots showed mild tail deviations and observed-versus-fitted plots displayed clustering near the LLQ threshold. All diagnostic plots are presented in Fig. S1.

### Sensitivity analyses

Baseline AMH was available for 317 women. Compared with those lacking baseline AMH, women with baseline AMH differed in age (*P* = 0.018), treatment group distribution (*P* = 0.004) and had shorter median follow-up (5.7 vs. 27.1 months; *P* < 0.001), whereas BMI, chemotherapy exposure, concurrent GnRHa use during chemotherapy, cyclophosphamide-based regimens, anti-HER2 therapy, and the number of AMH measurements per patient were comparable (Table S6). Therefore, the baseline-adjusted analysis was interpreted as a sensitivity analysis.

Among women with baseline AMH, the overall trajectory pattern remained consistent with the primary analysis: lower early predicted AMH levels in the CT/GnRHa-ET group, followed by progressive attenuation of between-group differences over time (Fig. S2). The baseline-AMH-adjusted sensitivity analysis included 317 patients and 767 AMH measurements, with follow-up density mainly within the first 48 months.

Exclusion of 17 observations with |standardized residuals|> 3 did not alter the estimated trajectory patterns relative to the primary model (Fig. S3). To evaluate potential selection bias related to repeated AMH testing, we compared patients with one AMH measurement and those with more than one measurement. Patients with repeated AMH testing were more likely to receive GnRHa-based endocrine therapy, but first AMH level, time to first AMH measurement, baseline AMH availability, chemotherapy exposure, and the proportion with at least one below-quantification AMH value were not significantly different between the two groups (Table S7).

Additional exploratory analyses evaluated treatment heterogeneity related to endocrine therapy partner and chemotherapy components. Results were consistent with the primary analysis.

Among chemotherapy-treated patients, additional adjustment for chemotherapy-related variables yielded a significant group-by-time interaction (χ^2^ = 34.27, df = 4, *P* < 0.001).

In an exploratory analysis restricted to patients receiving GnRHa-based endocrine therapy and classified according to endocrine therapy partner, no significant difference was observed between GnRHa plus AI and GnRHa plus SERM trajectories (χ^2^ = 4.59, df = 4, *P* = 0.332).

In a sensitivity analysis restricted to patients with at least three AMH measurements within 60 months (220 patients; 1070 observations), the group-by-time interaction remained significant, and the overall trajectory patterns were consistent with the primary analysis (χ^2^ = 157.33, df = 12, *P* < 0.001).

## Discussion

In this longitudinal real-world cohort of young women with early breast cancer, we observed distinct time-dependent AMH trajectories according to chemotherapy exposure and subsequent GnRHa-based endocrine therapy. Among chemotherapy-treated patients, women receiving GnRHa-based endocrine therapy had lower predicted AMH levels at 12 and 36 months than women not receiving GnRHa-based endocrine therapy, followed by attenuation of between-group differences at later time points. In contrast, among women who did not receive chemotherapy, GnRHa-based endocrine therapy was not associated with significant differences in predicted AMH. These findings indicate that AMH during GnRHa exposure represents a dynamic biomarker influenced by treatment context rather than a static measure of irreversible ovarian reserve loss.

The marked early AMH decline observed after chemotherapy is consistent with prior studies showing rapid ovarian reserve impairment after gonadotoxic treatment in young women with breast cancer and other cancers [6, 8–13]. AMH recovery after chemotherapy is known to vary according to age, baseline ovarian reserve, chemotherapy regimen, and follow-up timing [6, 8, 12, 13]. Our study extends these observations by modeling AMH continuously over time using repeated real-world measurements and flexible spline-based trajectories. This approach allowed us to capture nonlinear patterns that may be difficult to detect using single time-point comparisons or models assuming linear decline.

A key contribution of this study is the characterization of AMH dynamics during GnRHa-based endocrine therapy after breast cancer treatment. Prior randomized trials and meta-analyses have mainly evaluated GnRHa administered concurrently with chemotherapy for ovarian function preservation, focusing on premature ovarian insufficiency, menstrual recovery, pregnancy, or short-term AMH changes [17–20, 23–26]. In contrast, the longitudinal AMH pattern during subsequent adjuvant GnRHa-based endocrine therapy has been less clearly defined. Our findings indicate that early AMH values measured during GnRHa-based endocrine therapy were lower than those observed in patients not receiving GnRHa-based endocrine therapy, particularly after chemotherapy. However, this early difference diminished over time, suggesting that early AMH suppression during GnRHa exposure should not be interpreted in isolation.

These findings do not contradict prior randomized trials evaluating GnRHa during chemotherapy for ovarian function preservation. In our study, concurrent GnRHa use during chemotherapy was non-random, and the primary exposure of interest was subsequent GnRHa-based endocrine therapy rather than temporary ovarian suppression during chemotherapy.

These results have practical implications for survivorship care and fertility counseling. In young breast cancer survivors receiving pharmacologic ovarian suppression, a single low AMH value may reflect a combination of chemotherapy-related injury, age-related decline, and treatment-related suppression of follicular activity. Therefore, clinicians should be cautious in using isolated AMH measurements during GnRHa-based endocrine therapy to counsel patients about future reproductive potential. Interpreting AMH in relation to treatment phase, chemotherapy exposure, and longitudinal change may help avoid premature fertility pessimism and support more individualized discussions regarding ovarian function monitoring, endocrine therapy adherence, and future pregnancy planning [3, 4].

The biological interpretation of AMH changes during GnRHa exposure remains complex. GnRHa suppresses the hypothalamic-pituitary-ovarian axis and may alter follicular development, which can reduce AMH secretion from granulosa cells in growing follicles [7, 21, 22]. Thus, lower early AMH during GnRHa-based endocrine therapy may partly represent pharmacologic suppression rather than irreversible depletion of the primordial follicle pool. Preclinical and translational studies have proposed additional mechanisms by which GnRHa may reduce chemotherapy-induced ovarian injury, including modulation of endoplasmic reticulum stress, autophagy, and pathways related to follicle activation [31–33]. However, our data cannot directly distinguish transient endocrine suppression from true preservation of primordial follicles. Therefore, the observed attenuation of between-group differences over time should be interpreted as a longitudinal biomarker pattern rather than definitive evidence of direct ovarian protection.

Our study has several strengths. It included a relatively large cohort of young breast cancer survivors with repeated AMH measurements over extended follow-up. The use of linear mixed-effects models with restricted cubic splines allowed flexible modeling of nonlinear AMH trajectories while accounting for within-patient correlation from repeated measurements. We also evaluated the stability of long-term estimates by summarizing follow-up observation density, performed model diagnostic assessments, and conducted sensitivity analyses among women with available baseline AMH and after exclusion of extreme residual observations.

Several limitations should be acknowledged. First, this was a retrospective single-center study, and AMH measurements were obtained as part of routine clinical care rather than at protocol-mandated intervals. Therefore, testing frequency and timing may have been influenced by clinical indication, fertility concerns, or patient preference. Serial AMH testing was performed according to routine clinical practice rather than a predefined protocol, resulting in heterogeneous follow-up schedules (median of two measurements per patient). Nevertheless, sensitivity analyses among patients with more intensive longitudinal sampling yielded consistent trajectory patterns. Second, baseline AMH was not available for all patients, and patients with baseline AMH had different treatment group distribution and shorter follow-up. Although baseline-adjusted sensitivity analyses showed broadly consistent trajectory patterns, these analyses cannot fully eliminate selection bias. Information on oral contraceptive use was not systematically available and therefore could not be incorporated in the analysis. Third, AMH is a surrogate biomarker. We did not have direct fertility outcomes, including menstrual recovery, premature ovarian insufficiency, time to conception, pregnancy, or live birth. Fourth, the timing of GnRHa discontinuation and post-treatment ovarian function recovery was not standardized, limiting our ability to separate pharmacologic suppression from post-cessation recovery. Finally, despite adjustment for clinically relevant covariates, residual confounding by treatment indication, disease biology, or unmeasured reproductive factors remains possible. The consistency of findings in patients with more intensive longitudinal sampling supports the robustness of the primary trajectory analysis.

## Conclusion

In this longitudinal cohort of young women with breast cancer, AMH trajectories during GnRHa-based endocrine therapy were time-dependent and differed according to chemotherapy exposure. Among chemotherapy-treated patients, GnRHa-based endocrine therapy was associated with lower early predicted AMH levels followed by attenuation of between-group differences over time, whereas no significant difference was observed among patients without chemotherapy. These findings highlight that AMH measurements during GnRHa exposure should be interpreted according to treatment context and longitudinal patterns rather than as isolated indicators of ovarian reserve. Prospective studies incorporating protocol-mandated serial AMH measurements, ovarian function assessment after treatment discontinuation, and pregnancy or live birth data are needed to clarify the relationship between AMH dynamics and clinically meaningful reproductive outcomes.

## Supplementary Information

Below is the link to the electronic supplementary material.

Supplementary Material 1: Figure S1. Diagnostic plots for the primary multivariable linear mixed-effects model. A Residuals versus fitted values. B Quantile-quantile plot of residuals. C Residuals versus time since diagnosis. D Observed versus fitted lnAMH values

Supplementary Material 2: Figure S2. Baseline-AMH-adjusted sensitivity analysis of longitudinal AMH trajectories (317 patients; 767 AMH measurements). Follow-up density declined over time, with 31 unique patients contributing 55 AMH measurements during the 36–60-month interval; estimates at later time points should therefore be interpreted cautiously

Supplementary Material 3: Figure S3. Sensitivity analysis of longitudinal AMH trajectories after exclusion of observations with extreme standardized residuals (612 patients; 1,585 AMH measurements). Sensitivity analysis excluding 17 observations with absolute standardized residuals >3 from the primary complete-case model

Supplementary Material 4

Supplementary Material 5

Supplementary Material 6

Supplementary Material 7

Supplementary Material 8

Supplementary Material 9

Supplementary Material 10

## Data Availability

The data underlying this article will be shared upon reasonable request to the corresponding author.

## Abbreviations

AI: Aromatase inhibitor
AMH: Anti-Müllerian hormone
BMI: Body mass index
CT: Chemotherapy
DCIS: Ductal carcinoma in situ
EBC: Early breast cancer
ET: Endocrine therapy
GnRHa: Gonadotropin-releasing hormone agonist
HER2: Human epidermal growth factor receptor 2
IQR: Interquartile range
LCIS: Lobular carcinoma in situ
LLQ: Lower limit of quantification
SERM: Selective estrogen receptor modulator
SYSUCC: Sun Yat-sen University Cancer Center
