# Supplementary material for: Time-dependent anti-Müllerian hormone dynamics during GnRHa-based endocrine therapy in young breast cancer survivors: a longitudinal real-world cohort study
Source: Breast Cancer Res. 2026 Aug 4;28:185. doi: 10.1186/s13058-026-02359-9 (PMC13721651; doi:10.1186/s13058-026-02359-9)
Supplement: Supplementary file 1 [file 13058_2026_2359_MOESM10_ESM.docx]

**Table S7. Characteristics according to AMH testing frequency.**

| Characteristic | One AMH measurement  (N = 291) | More than one AMH measurement  (N = 394) | P |
| --- | --- | --- | --- |
| Age at diagnosis, years | 35 (32, 37) | 35 (32, 37) | 0.450 |
| BMI category |  |  | 0.113 |
| <25 kg/m^2^ | 235 (83.3) | 333 (87.9) |  |
| ≥25 kg/m^2^ | 47 (16.7) | 46 (12.1) |  |
| Missing | 9 | 15 |  |
| Treatment group |  |  | 0.007 |
| No CT/No GnRHa-ET | 54 (18.6) | 81 (20.6) |  |
| No CT/GnRHa-ET | 22 (7.6) | 38 (9.6) |  |
| CT/No GnRHa-ET | 108 (37.1) | 98 (24.9) |  |
| CT/GnRHa-ET | 107 (36.8) | 177 (44.9) |  |
| Chemotherapy exposure |  |  | 0.266 |
| No CT | 76 (26.1) | 119 (30.2) |  |
| CT | 215 (73.9) | 275 (69.8) |  |
| GnRHa-based endocrine therapy |  |  | 0.009 |
| No GnRHa-ET | 162 (55.7) | 179 (45.4) |  |
| GnRHa-ET | 129 (44.3) | 215 (54.6) |  |
| Baseline AMH available |  |  | 0.757 |
| No | 154 (52.9) | 214 (54.3) |  |
| Yes | 137 (47.1) | 180 (45.7) |  |
| Time to first AMH measurement, months | 3.5 (0, 11.8) | 3.7 (0, 13.1) | 0.863 |
| First AMH, ng/mL | 1.06 (0.02, 2.9) | 1.1 (0.19, 3.02) | 0.164 |
| First AMH below LLQ |  |  | 0.027 |
| No | 244 (83.8) | 354 (89.8) |  |
| Yes | 47 (16.2) | 40 (10.2) |  |
| At least one AMH below LLQ |  |  | 0.231 |
| No | 244 (83.8) | 316 (80.2) |  |
| Yes | 47 (16.2) | 78 (19.8) |  |
| AMH follow-up span, months | 0 (0, 0) | 15.9 (7.9, 26.6) | <0.001 |
| Number of AMH measurements | 1 (1, 1) | 3 (2, 5) | <0.001 |

Values are shown as n (%) or median (interquartile range). Percentages were calculated using the number of patients with available data in each AMH testing-frequency group. P values were calculated using Fisher’s exact test or Wilcoxon rank-sum test, as appropriate; missing values were excluded from P-value calculation.
