# Supplementary material for: Time-dependent anti-Müllerian hormone dynamics during GnRHa-based endocrine therapy in young breast cancer survivors: a longitudinal real-world cohort study
Source: Breast Cancer Res. 2026 Aug 4;28:185. doi: 10.1186/s13058-026-02359-9 (PMC13721651; doi:10.1186/s13058-026-02359-9)
Supplement: Supplementary file 2 [file 13058_2026_2359_MOESM4_ESM.docx]

**Table S1 Chemotherapy characteristics and systemic treatment exposure among chemotherapy-treated patients.**

| **Characteristic** | **CT/No GnRHa-ET (N = 206)** | **CT/GnRHa-ET (N = 284)** | **P** |
| --- | --- | --- | --- |
| Chemotherapy components |  |  |  |
| Anthracycline-containing | 150 (72.8) | 241 (84.9) | 0.001 |
| Taxane-containing | 190 (92.2) | 257 (90.5) | 0.523 |
| Cyclophosphamide-containing | 153 (74.3) | 264 (93.0) | <0.001 |
| Carboplatin-containing | 54 (26.2) | 16 (5.6) | <0.001 |
| Chemotherapy cycles | 8 (6, 8) | 8 (6, 8) | 0.129 |
| Neoadjuvant chemotherapy |  |  | 0.023 |
| No | 117 (56.8) | 191 (67.3) |  |
| Yes | 89 (43.2) | 93 (32.7) |  |
| Immunotherapy |  |  | <0.001 |
| No | 147 (71.4) | 281 (98.9) |  |
| Yes | 59 (28.6) | 3 (1.1) |  |
| Concurrent GnRHa during chemotherapy |  |  | 0.001 |
| No | 143 (69.4) | 170 (59.9) |  |
| Yes | 35 (17.0) | 87 (30.6) |  |
| Missing | 28 (13.6) | 27 (9.5) |  |
| Anti-HER2 therapy |  |  | 0.030 |
| No | 140 (68.0) | 219 (77.1) |  |
| Yes | 66 (32.0) | 65 (22.9) |  |

Values are shown as n (%) or median (interquartile range). Percentages were calculated using the total number of patients in each treatment group. P values were calculated using Fisher’s exact test or Wilcoxon rank-sum test, as appropriate; missing values were excluded from P-value calculation.

Missing values reflect unavailable treatment records before referral to our institution.

CT, chemotherapy; ET, endocrine therapy; GnRHa, gonadotropin-releasing hormone agonist; HER2, human epidermal growth factor receptor 2.
