# Supplementary material for: Time-dependent anti-Müllerian hormone dynamics during GnRHa-based endocrine therapy in young breast cancer survivors: a longitudinal real-world cohort study
Source: Breast Cancer Res. 2026 Aug 4;28:185. doi: 10.1186/s13058-026-02359-9 (PMC13721651; doi:10.1186/s13058-026-02359-9)
Supplement: Supplementary file 3 [file 13058_2026_2359_MOESM5_ESM.docx]

**Table S2. AMH measurements below the lower limit of quantification (LLQ).**

**A. Measurement-level summary**

| **Section** | Category | AMH measurements | Below LLQ, n (%) |
| --- | --- | --- | --- |
| Overall | Overall | 1758 | 240 (13.7) |
| By treatment group | CT/GnRHa-ET | 814 | 194 (23.8) |
| By treatment group | CT/No GnRHa-ET | 414 | 43 (10.4) |
| By treatment group | No CT/GnRHa-ET | 187 | 3 (1.6) |
| By treatment group | No CT/No GnRHa-ET | 343 | 0 (0) |
| By chemotherapy exposure | CT | 1228 | 237 (19.3) |
| By chemotherapy exposure | No CT | 530 | 3 (0.6) |
| By follow-up interval | 0-12 months | 789 | 120 (15.2) |
| By follow-up interval | 12-24 months | 408 | 79 (19.4) |
| By follow-up interval | 24-36 months | 212 | 28 (13.2) |
| By follow-up interval | 36-60 months | 211 | 13 (6.2) |
| By follow-up interval | 60-84 months | 74 | 0 (0) |
| By follow-up interval | 84-108 months | 43 | 0 (0) |
| By follow-up interval | ≥108 months | 21 | 0 (0) |

**B. Patient-level summary**

| **Section** | **Category** | **Patients** | **Patients with ≥1 below-LLQ AMH, n (%)** |
| --- | --- | --- | --- |
| Patient-level overall | Overall | 685 | 125 (18.2) |
| Patient-level by treatment group | CT/GnRHa-ET | 284 | 89 (31.3) |
| Patient-level by treatment group | CT/No GnRHa-ET | 206 | 35 (17) |
| Patient-level by treatment group | No CT/GnRHa-ET | 60 | 1 (1.7) |
| Patient-level by treatment group | No CT/No GnRHa-ET | 135 | 0 (0) |
| Patient-level by chemotherapy exposure | CT | 490 | 124 (25.3) |
| Patient-level by chemotherapy exposure | No CT | 195 | 1 (0.5) |

Values are numbers of AMH measurements or patients. AMH values below the lower limit of quantification (LLQ) were assigned 0.01 ng/mL for analysis. Some AMH results were reported by the clinical laboratory as 0.01 ng/mL rather than “<LLQ”. These values were retained as reported and therefore were not classified as below the lower limit of quantification.
