# Supplementary material for: Time-dependent anti-Müllerian hormone dynamics during GnRHa-based endocrine therapy in young breast cancer survivors: a longitudinal real-world cohort study
Source: Breast Cancer Res. 2026 Aug 4;28:185. doi: 10.1186/s13058-026-02359-9 (PMC13721651; doi:10.1186/s13058-026-02359-9)
Supplement: Supplementary file 4 [file 13058_2026_2359_MOESM6_ESM.docx]

**Table S3. Distribution of unique patients and AMH measurements contributing to the primary complete-case longitudinal trajectory analyses.**

| Time intervals | No CT/No GnRHa-ET | No CT/ GnRHa-ET | CT/No GnRHa-ET | CT/GnRHa-ET | All patients |
| --- | --- | --- | --- | --- | --- |
| 0-12 months | 104 / 164 | 43 / 77 | 120 / 163 | 192 / 323 | 459 / 727 |
| 12-24 months | 40 / 72 | 24 / 44 | 38 / 69 | 94 / 182 | 196 / 367 |
| 24-36 months | 24 / 32 | 15 / 22 | 26 / 41 | 59 / 98 | 124 / 193 |
| 36-60 months | 28 / 44 | 12 / 18 | 28 / 39 | 52 /92 | 120 / 193 |
| 60-84 months | 7 / 8 | 6 / 7 | 21 / 31 | 17 / 23 | 51 / 69 |
| 84-108 months | 6 / 8 | 1 / 2 | 10 / 11 | 13 / 15 | 30 / 36 |
| ≥ 108 months | 3 / 7 | 0 / 0 | 3 / 4 | 3 / 6 | 9 / 17 |
| Overall |  |  |  |  | 613 / 1,602 |

Overall patient counts represent unique patients across the entire analysis cohort and therefore are not equal to the sum of patient counts across follow-up intervals, because individual patients may contribute measurements to more than one interval.

Trajectories were truncated at 84 months because observations beyond this point were sparse and produced unstable estimates.
