# Supplementary material for: Time-dependent anti-Müllerian hormone dynamics during GnRHa-based endocrine therapy in young breast cancer survivors: a longitudinal real-world cohort study
Source: Breast Cancer Res. 2026 Aug 4;28:185. doi: 10.1186/s13058-026-02359-9 (PMC13721651; doi:10.1186/s13058-026-02359-9)
Supplement: Supplementary file 5 [file 13058_2026_2359_MOESM7_ESM.docx]

**Table S4 Pairwise comparisons of predicted AMH: No CT/No GnRHa-ET and** **No CT/GnRHa- ET.**

| Months | Estimate | Adjusted P |
| --- | --- | --- |
| 12 | +0.421 | 0.486 |
| 36 | +0.143 | 0.973 |
| 60 | -0.007 | 1.000 |
| 84 | -0.092 | 0.999 |

Estimate = lnAMH (No CT/No GnRHa-ET) − lnAMH(No CT/GnRHa- ET). A positive value indicates higher predicted lnAMH in Group No CT/No GnRHa-ET; a negative value indicates higher predicted lnAMH in Group No CT/GnRHa- ET.

Adjusted P values were obtained from post-hoc pairwise comparisons using Tukey’s method.

Abbreviations: CT, chemotherapy; ET, endocrine therapy; GnRHa, gonadotropin-releasing hormone agonist.
