# Supplementary material for: Time-dependent anti-Müllerian hormone dynamics during GnRHa-based endocrine therapy in young breast cancer survivors: a longitudinal real-world cohort study
Source: Breast Cancer Res. 2026 Aug 4;28:185. doi: 10.1186/s13058-026-02359-9 (PMC13721651; doi:10.1186/s13058-026-02359-9)
Supplement: Supplementary file 6 [file 13058_2026_2359_MOESM8_ESM.docx]

**Table S5. Pairwise comparisons of predicted AMH: CT/No GnRHa-ET and** **CT/GnRHa-ET.**

| Months | Estimate | Adjusted P |
| --- | --- | --- |
| 12 | +0.566 | 0.026 |
| 36 | +0.644 | 0.023 |
| 60 | +0.435 | 0.267 |
| 84 | +0.113 | 0.988 |

Estimate = lnAMH (CT/No GnRHa-ET) − lnAMH(CT/GnRHa-ET). A positive value indicates higher predicted lnAMH in CT/No GnRHa-ET; a negative value indicates higher predicted lnAMH in CT/GnRHa-ET.

Adjusted P values were obtained from post-hoc pairwise comparisons using Tukey’s method.

Abbreviations: CT, chemotherapy; ET, endocrine therapy; GnRHa, gonadotropin-releasing hormone agonist.
