# Supplementary material for: Time-dependent anti-Müllerian hormone dynamics during GnRHa-based endocrine therapy in young breast cancer survivors: a longitudinal real-world cohort study
Source: Breast Cancer Res. 2026 Aug 4;28:185. doi: 10.1186/s13058-026-02359-9 (PMC13721651; doi:10.1186/s13058-026-02359-9)
Supplement: Supplementary file 7 [file 13058_2026_2359_MOESM9_ESM.docx]

**Table S6. Characteristics of patients according to baseline AMH availability.**

| Characteristic | No baseline AMH (N = 368) | Baseline AMH available (N = 317) | P |
| --- | --- | --- | --- |
| Group |  |  | 0.004 |
| No CT/No GnRHa-ET | 58 (15.8) | 77 (24.3) |  |
| No CT/GnRHa-ET | 42 (11.4) | 18 (5.7) |  |
| CT/No GnRHa-ET | 117 (31.8) | 89 (28.1) |  |
| CT/GnRHa-ET | 151 (41.0) | 133 (42.0) |  |
| Age (Median, IQR, years) | 35.0 (31.8, 37.0) | 35.0 (32.0, 38.0) | 0.018 |
| BMI (kg/m^2^) |  |  | 0.911 |
| <25 | 299 (86.2) | 269 (85.7) |  |
| ≥25 | 48 (13.8) | 45 (14.3) |  |
| Missing | 21 | 3 |  |
| Chemotherapy |  |  | 0.445 |
| No | 100 (27.2) | 95 (30.0) |  |
| Yes | 268 (72.8) | 222 (70.0) |  |
| Concurrent GnRHa during chemotherapy |  |  | 0.364 |
| No | 267 (82.2) | 241 (79.0) |  |
| Yes | 58 (17.8) | 64 (21.0) |  |
| Missing | 43 | 12 |  |
| Cyclophosphamide-based chemotherapy |  |  | 0.307 |
| No | 137 (37.2) | 131 (41.3) |  |
| Yes | 231 (62.8) | 186 (58.7) |  |
| Anti-HER2 therapy |  |  | 1.000 |
| No | 298 (80.8) | 256 (80.8) |  |
| Yes | 70 (19.2) | 61 (19.2) |  |
| AMH tests per patient (Median, IQR, times) | 2 (1,4) | 2 (1,3) | 0.088 |
| Follow-up (Median, IQR, months) | 27.1 (10.8, 55.5) | 5.7 (0, 17.9) | <0.001 |

Values are median, IQR or N, %.

Baseline AMH was defined as an AMH measurement obtained before initiation of systemic anticancer therapy.

Percentages for variables with missing data were calculated among patients with available data; missing values were excluded from P-value calculations.

AMH, anti-Müllerian hormone; BMI, body mass index; CT, chemotherapy; GnRHa, gonadotropin-releasing hormone agonist; ET, endocrine therapy; HER2, human epidermal growth factor receptor 2.
